# Larger Medicine

**Published:** 1908-10-24

**Authors:** 


The Hospital

A Journal of

The Medical Sciences and Hospital Administration.

New Series. No. 85, Vol. IV. [No. 1157, Vol. XLV.] SATURDAY, OCTOBER 24, 1908.

### Larger Medicine.

The introductory address delivered by Sir Thomas Clifford Allbutt to the University Medical School of Manchester maintains the high level both of manner and matter which Sir Clifford has taught us to look for in his utterances and writings. The speech referred to was concerned, in the first instance, with some of the crucial problems of hospital management, especially the hitherto unsolved problem of hospital out-patient departments. This has, of course, been a stone of offence to the general practitioner for years, the thesis of the latter being that a large proportion of those who avail themselves of hospital treatment for trifling disorders have no proper title to the service of a charity, and that it is an inequitable business to drive the general practitioner into competition of this kind. No doubt the matter is excessively difficult, for, in the lower spheres of income particularly, the actual receipts of a man, without a knowledge of his outgoings, give no information as to his title to charitable treatment in case of illness. Moreover, who shall say that this or that complaint is trifling because physical examination is negative? It is a commonplace of medicine that it is easier to diagnose a disease than to be confident that none exists; indeed, no man who took thought for his reputation would risk it on such a presumption except after a most rigorous examination and much thought, and these two processes are unluckily incompatible with the service of a crowded out-patient department. Sir Clifford Allbutt suggests that something might be done to bring the general practitioner and the hospital staff together, to the benefit of all concerned, by establishing regular hours of free consultation during which any practitioner might bring to the hospital such of his poorer patients as presented peculiar difficulty in diagnosis, or require costly therapeutic appliances for their proper treatment. In order to be of real service these hours of consultation would need to be invariable and frequently recurring, the hospital giving an undertaking that any practitioner bringing a patient at these hours should be able to rely upon a consultation with a representative of the honorary staff. In this way a patient who, though unable to pay a consultation-fee, is able to pay his regular medical attendant, would not be driven to cut himself adrift from the latter the moment he, or his doctor for that matter, wishes for a second opinion. We commend this scheme to the managers of our hospitals. It will not solve the out-patient problem. That is the growth of years and has already assumed the proportions of a national question; but it will tend to a harmonious collaboration between two large groups of public servants, namely, general practitioners and hospital staffs, a collaboration which cannot but reflect itself in advantage to the public.

In the latter part of his speech Sir Clifford was concerned with the relation between medicine and the State. In the course of a thoughtful survey of the present status of the medical profession and its probable course of evolution, he observed that until recent years medicine has lacked the consideration and dignity accorded to the Church, the law, the army, and the navy. The explanation is to be found in the fact that the concerns of medicine have so far been individual, and as such have had no claim to public recognition. It is only when medicine deals with the larger issues of public health that she becomes of national importance; and in this respect medicine has only recently acquired the knowledge which would justify her in assuming responsibility. What is now required in England is the establishment of a general staff of medicine "to rebuke the purblind and inveterate habit of our countrymen of devoting their magnificent energy and their treasure in mopping up effects in disregard of causes."

In this pregnant sentence Sir Clifford Allbutt indicates a weakness unworthy of our boasted reputation as a practical nation. We have alluded to it in these columns on previous occasions, but make no apology for condemning it again, so obviously foolish and perverse is it: we mean the unhappy faculty of preferring cure to prevention. We pour out money like water in order to found sanatoria for consumptives, asylums for the insane, prisons for the criminal, yet are moved with the utmost difficulty to subscribe to the agencies which study to prevent these evils. The fault lies primarily in the uncoordinated state of medicine deplored by Sir Clifford. At present it is nobody's business to educate the public in those rules of life and living which are the springs of national efficiency. Here a private body, there an enthusiastic medical officer of health, and everywhere practitioners of medicine in their limited fields attempt to sow the seeds of this vital knowledge, but the thing is done in a random, haphazard way quite unworthy of its importance. There can be little doubt that the near future will see the establishment of a ministry of public health, for the functions of medicine are rapidly becoming too all-pervading to be any longer neglected by the " unidea'd governing classes," as Professor Allbutt calls them. How all-pervading these functions are is hardly realised even by our own profession. Here are some of the more important of those instanced in the address before us. The protection of infant life; the medical inspection of school children; the physical and mental conditions of education; the conditions of labour, the dynamics of food, and the minimum wage; factory inspection, with estimates of the effects of different trades upon health, and collateral problems of compensation; housing, ventilation, and sanitation; food markets and adulteration; epidemic diseases; the working of the Poor Law; the campaign against drunkenness, fornication, and other social vices; criminology and punishment, and the " antiquated and distrusted dogmas of the judges of the higher courts on responsibility before the law." Here, surely, is enough to justify the establishment of a formal head to control and co-ordinate the usefulness of so many members; yet the matter requires caution in the handling, lest by premature and excessive centralisation of authority the vitality and initiative of the members be impaired. An immense and beneficent work will lie before a sympathetic and large-minded minister of public health.

